# Chronic and moderate consumption of reduced-alcohol wine confers cardiac benefits in a rat model of pulmonary arterial hypertension

**DOI:** 10.1186/s13104-021-05738-x

**Published:** 2021-08-23

**Authors:** Patrick Diaba-Nuhoho, Martin Cour, Nkanyiso Hadebe, David Marais, Sandrine Lecour, Dee Blackhurst

**Affiliations:** 1 Division of Chemical Pathology, Department of Pathology, University of Cape Town, Cape Town, South Africa; 2 Cardioprotection Group, Hatter Cardiovascular Institute and Lionel Opie Preclinical Imaging Core Facility, University of Cape Town, Cape Town, South Africa; 3 Department of Anaesthesia, Groote Schuur Hospital and University of Cape Town, Cape Town, South Africa; 4 Division of Vascular Endothelium and Microcirculation, Department of Medicine III, University of Technology Dresden, Fetscherstr. 74, 01307 Dresden, Germany

**Keywords:** Antioxidants, Monocrotaline, Oxidative stress, Pulmonary arterial hypertension, Red wine

## Abstract

**Objectives:**

In pulmonary arterial hypertension (PAH), right ventricular (RV) dysfunction develops via mechanisms involving oxidative stress. Moderate and chronic red wine (RW) consumption reduces oxidative stress and confers cardioprotection but its effect on PAH is unknown. We evaluated whether moderate and chronic consumption of reduced-alcohol RW (RARW) confers cardioprotection in a monocrotaline (MCT)-induced PAH rat model.

**Results:**

Rats were randomly grouped: control; MCT; RARW; MCT + RARW. Wine was diluted to mimic moderate intake for humans, and consumed from 7 days before, until 28 days after MCT-injection. Echocardiography measured pulmonary artery acceleration time (PAAT) and RV thickness. Conjugated dienes (CD), and thiobarbituric acid reactive substances (TBARS) concentrations were assessed. MCT induced RV thickness and decreased PAAT compared to controls [1.22 ± 0.09 mm vs 0.46 ± 0.02 mm and 14 ± 1 vs 23 ± 2 m/s, respectively (p < 0.001)]. Chronic RARW consumption limited MCT-induced RV hypertrophy and increased PAAT. CD and TBARS increased in MCT-treated animals compared to controls (672 ± 43 nmol/L vs 453 ± 35 nmol/L; p < 0.01 and 13 ± 2 µmol/L vs 4 ± 0.3 µmol/L; p < 0.01). RARW reduced MCT-induced CD (472 ± 27 nmol/L vs 672 ± 43 nmol/L; p < 0.01).

**Conclusion:**

Chronic and moderate intake of RARW ameliorates MCT-induced PAH in rats, which may be partly attributable to reduction of lipid peroxidation.

**Supplementary Information:**

The online version contains supplementary material available at 10.1186/s13104-021-05738-x.

## Introduction

Pulmonary arterial hypertension (PAH) is a disease with high morbidity and mortality [1]. All PAH-related diseases share common pathological features characterised by inflammation, pulmonary vascular remodelling, vasoconstriction, thrombosis in situ and right ventricular (RV) hypertrophy [2]. There is no cure for PAH, and current medical treatments are limited due, at least in part, to the lack of knowledge of the pathophysiology of the disease. Recent data suggest that oxidative stress may contribute to pulmonary vascular dysfunction, therefore supporting the hypothesis that the use of antioxidants may be beneficial as a therapeutic approach for the disease [2, 3].

Multiple epidemiological and experimental studies suggest that moderate and chronic consumption of red wine confers health benefits, an effect that can be attributed to both alcohol and other compounds in wine [4, 5]. Indeed, lowering the alcohol levels in wine does not seem to affect the cardioprotective effect of regular and moderate consumption of wine against ischemia–reperfusion injury but it presents the beneficial effect of potentially limiting the adverse effects of alcohol [4]. In humans, moderate consumption of wine may decrease the risk of chronic obstructive pulmonary disease [6]. Although several components found in wine such as melatonin, resveratrol and quercetin present health benefits against PAH in animal studies, it is not known whether moderate and chronic consumption of wine protects against PAH [7–9]. Therefore, in the present study, we aimed to test whether chronic and moderate consumption of reduced-alcohol RW (RARW) could limit monocrotaline (MCT)-induced PAH in rats.

## Main text

### Methodology

#### Animal group

The study was approved by the Faculty of Health Sciences Animal Ethics Committee, University of Cape Town (under the reference number 016/001) and conducted at the Hatter Institute. All protocols were carried out in compliance with the Care and Use of Laboratory Animals Guide published by the United States National Institutes of Health in 2011. Male Long Evans rats obtained from the Research Animal Facility, University of Cape Town (RAF-UCT) weighing 150–175 g were housed in the Faculty of Health Sciences Animal Unit at the University of Cape Town, in a 12-h dark/light cycle with room temperatures between 20 and 23 °C and humidity maintained at 40%. A maximum of 4 rats were housed per cage. The drinking water of the rats was supplemented with one part of 5.5% reduced-alcohol red wine diluted to seven parts of water, mimicking an amount equivalent to approximately 1–2 glasses of wine per day in humans, as previously described [4, 10]. The groups were pre-treated with wine or water for 7 days before receiving a single injection of physiological saline (0.9%, 0.1 mL) or MCT (80 mg/kg subcutaneous, 0.1 mL), see Additional file 1: Figure S1.

The following randomised groups of rats were used: control rats (C) (n = 5) received only water; MCT rats (n = 7) received water and an injection of MCT; RARW rats (n = 5) received reduced-alcohol wine; MCT + RARW rats (n = 7) received RARW and an injection of MCT. The total duration of the study was 35 days, which included the 7 days of pre-treatment with RARW or water, and 28 days following the MCT injection. Echocardiography was performed on all the rats on day -7 and again on day 28, see Additional file 1: Figure S1.

#### Blood sample collection and euthanasia

At 28 days, deep unconsciousness was induced using 40 mg/kg of Sodium Pentobarbital (intraperitoneally) mixed with phosphate buffered saline (PBS) after anesthetising with isoflurane. Blood samples of approximately 6 mL each were drawn by cardiac puncture, after thoracotomy using a 25G needle into a serum gel separator tube and an EDTA anticoagulant tube as previously described [8]. Sample tubes were gently mixed and centrifuged for 15 min at 2500×*g*. Serum and plasma were stored in aliquots at − 80 °C until analysed. Once the blood was collected, the heart was excised from the chest cavity for the biometric measurements.

#### Biometric measurements

Hearts and livers were removed, rinsed with PBS, lightly dried with a paper tissue and weighed. The RV was separated from the LV plus septum, and their masses were recorded. The length of the right tibia of every rat was also recorded. The ratios of the mass of the RV to the LV plus septum, to tibia length and to the whole heart were calculated and used for different indices of RV hypertrophy [8]. Relative changes of liver mass were determined by dividing the mass of the liver by the body mass [11].

#### Echocardiography

At day-7 and at the end of the study (total of 35 days), the rats were slightly anesthetised with 1.5–2% isoflurane and positioned in the supine position on a warming pad. Closed chest high-resolution echocardiography was performed with a VEVO 2100 ultrasound system (Fujifilm, Visualsonics, Ontario, Canada) and a 13–25 MHz linear array transducer. LV chamber size and ejection fraction were obtained from 2 dimensional and M-mode measurements at the mid-papillary level. To assess LV diastolic function, both mitral E and A wave peak velocities were obtained from pulse-wave Doppler in the apical 4-chamber view; the E/A ratio was then calculated. From pulse-wave Doppler tracing of pulmonary outflow in the parasternal view at the level of the aortic valve, pulmonary artery acceleration time (PAAT) was measured as the flow time from start to peak velocity and normalised to total RV ejection time; velocity–time integration (VTI) was also calculated. PAAT/ ejection time ratio was used as a surrogate marker of PAH [12]. Tricuspid annular plane systolic excursion (TAPSE), a marker of systolic RV function, was obtained from the apical 4-chamber view. Finally, to assess RV hypertrophy, diastolic thickness of the RV free wall was measured. All measurements were made off-line on the mean of at least three consecutive cardiac cycles with the software resident on the ultrasound system.

#### Histology of hearts

The RV as well as the LV plus septum were fixed in buffered 4% formalin, embedded in paraffin and sectioned longitudinally at a thickness of 5 µm for histological staining and analysis. Sections were examined microscopically with a Nikon Eclipse 90i microscope using a 20X objective. Images were captured and analysed using ImageJ software (https://imagej.nih.gov/ij/index.html) and data expressed as percentages of collagen deposition per total area sectioned [13].

#### Antioxidant and oxidant analyses

##### Blood plasma superoxide dismutase (SOD) and catalase activity determination

The SOD activity assay was measured as preciously described by McCord and Fridovich [14]. The rate of reduction was measured spectrophotometrically at 550 nm over 5 min intervals from time zero with a microplate data acquisition program (SoftMax® Pro 4.8). The final reaction slope was determined from the use of a SOD standard curve and the SOD activity expressed as international units per milligram protein (IU/mg protein). Catalase activity was evaluated by the reduction of H_2_O_2_ using the method of Aebi [15]. Plasma samples were diluted 1:10 in 50 mmol/L phosphate buffer, pH 7.0. The diluted samples (10μL) were added to a Costar® (Corning) 96-well, UV-transparent plate (Sigma-Aldrich, SA), with 10 μL of 50 mM phosphate buffer, pH 7.0 and 220μL of a freshly prepared solution of 40 mmol/L (from 30%) H_2_O_2_. The rate of decomposition of H_2_O_2_ was measured spectrophotometrically (SPECTRAmaxPLUS-384) from changes in absorbance at 240 nm over 5 min, relative to a blank (20 μL phosphate buffer plus 220 μL of the 40 mmol/L H_2_O_2_). The activity of catalase was expressed as international units/mg protein using a molar extinction coefficient of H_2_O_2_ at 240 nm = 43.6 M^−1^ cm^−1^.

##### Plasma concentration of lipid peroxidation products (TBARS and CD)

Lipid peroxidation was determined by measuring malondialdehyde (MDA), a lipid peroxidation product which is measured by the thiobarbituric acid reactive substances (TBARS) assay method of Asakawa and Matsushita [16]. Conjugated dienes (CD), as initial markers of lipid peroxidation, were measured in cyclohexane at a wavelength of 234 nm after an initial Folch extraction of the plasma [17].

#### Statistical analysis

The data obtained were analysed using GraphPad Prism version 6.00 for windows, GraphPad software (La Jolla, CA USA). ANOVA was used for comparing several groups. Where significant differences were observed using the two-way ANOVA, post-hoc Bonferroni’s test was used to evaluate differences within groups. The data were reported as mean ± standard error of the mean (SEM). Statistical significance was set at p < 0.05.

### Results

#### Biometric measurements

At the end of the experiments, all groups had similar body mass, heart mass and liver mass as shown in Table 1. A single subcutaneous injection of MCT increased the RV mass, RV mass/tibia length, the RV/(LV + septal mass) and decreased (LV + septal mass)/heart mass compared to the control group, (p < 0.05). Chronic and moderate consumption of RARW given in rats receiving MCT, significantly decreased RV mass/(LV + septal mass) and increased the (LV + septal mass)/heart mass compared to MCT alone (p < 0.05).

**Table 1:** The effect of RARW consumption on biometric measurements in rats

	C	MCT	RARW	MCT + RARW
Body mass (g)	311.9 ± 21.6	292.6 ± 4.2	291.0 ± 16.3	283.9 ± 12.6
Whole heart mass (g)	1.59 ± 0.11	1.65 ± 0.08	1.44 ± 0.07	1.47 ± 0.10
RV mass (g)	0.23 ± 0.03	0.39 ± 0.03*	0.23 ± 0.04^†^	0.29 ± 0.04
Liver mass (g)	18.18 ± 1.30	17.29 ± 0.51	17.49 ± 0.62	17.13 ± 0.89
Tibia length (cm)	4.80 ± 0.20	4.81 ± 0.07	5.02 ± 0.02	4.64 ± 0.12
LV + septal mass (g)	0.93 ± 0.04	0.86 ± 0.03	0.91 ± 0.03	0.89 ± 0.06
(LV + septal mass)/heart mass (g/g)	0.59 ± 0.02	0.52 ± 0.01*	0.64 ± 0.02	0.61 ± 0.02^†^
Heart mass/tibia length (g/cm)	0.33 ± 0.03	0.34 ± 0.02	0.29 ± 0.01	0.32 ± 0.02
RV/(LV + septal mass) (g/g)	0.24 ± 0.03	0.45 ± 0.02*	0.25 ± 0.03	0.32 ± 0.03^†^
RV/tibia length (g/cm)	0.047 ± 0.007	0.081 ± 0.006*	0.045 ± 0.007^†^	0.063 ± 0.008
Heart mass/body mass (g/g)	0.0052 ± 0.0008	0.0057 ± 0.0003	0.0050 ± 0.0002	0.0052 ± 0.0004
Liver mass/body mass (g/g)	0.061 ± 0.009	0.059 ± 0.002	0.060 ± 0.002	0.060 ± 0.001

Rats consumed wine for 7 days before and 28 days after being injected with PAH-inducing MCT

Values are means ± S.E.M

*C* Control, *MCT* monocrotaline, *RARW* reduced-alcohol red wine, *RV* right ventricular, *LV* left ventricular

*p < 0.05 (vs C); ^†^p < 0.05 (vs MCT); n ≥ 5 per group

#### Echocardiography measurements

**Fig. 1:**
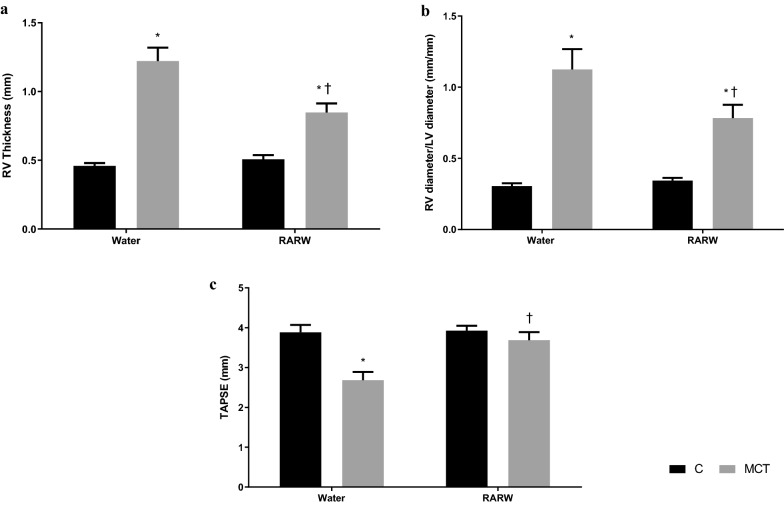
The effect of chronic, moderate and reduced-alcohol red wine on biometric and cardiac parameters. Rats were divided into 4 groups: those who consumed water (Control), reduced-alcohol wine (RARW) and/or those who received an injection of PAH-inducing MCT. All rats consumed the respective beverages for 7 days before until 28 days after the MCT injection. **a** Effect of moderate, chronic treatment of RARW on the right ventricular free wall in MCT-induced PAH. **b** Effect of moderate, chronic treatment of RARW on the ratio of the RV diameter to LV diameter in MCT induced PAH. **c** Effect of moderate, chronic treatment of RARW on the Tricuspid Annular Plane Systolic excursion in MCT induced PAH. *C* Control, *MCT* Monocrotaline, *RARW* Reduced-alcohol red wine, *RV* Right ventricle. Values are mean ± S.E.M. *p < 0.05 (vs C); ^†^p < 0.05 (vs MCT); n ≥ 5 per group

In Fig. 1, MCT increased the RV thickness, the RV/LV diameter ratio and decreased the TAPSE (p < 0.05 versus control). Chronic and moderate consumption of RARW significantly limited RV hypertrophy and dysfunction (p < 0.05 versus MCT). In Table 2, MCT induced PAH as demonstrated by a decrease in PAAT, PAAT/ejection time, peak velocity and VIT (p < 0.05 versus control). Chronic and moderate treatment of RARW partially reversed MCT-induced PAH (p < 0.05 versus MCT).

**Table 2:** Echocardiography measurements 28 days after MCT injection

	C	MCT	RARW	MCT + RARW
Heart rate (bpm)	414 ± 26	362 ± 22	415 ± 39	397 ± 18
Left ventricle				
IVS, diastole (mm)	1.75 ± 0.08	1.56 ± 0.23	1.61 ± 0.06	1.59 ± 0.09
IVS, systole (mm)	3.08 ± 0.14	2.85 ± 0.30	2.82 ± 0.07	2.75 ± 0.27
LVID, diastole (mm)	8.28 ± 0.32	7.90 ± 0.32	8.19 ± 0.29	8.12 ± 0.34
LVID, systole (mm)	4.98 ± 0.41	4.86 ± 0.46	5.02 ± 0.27	4.80 ± 0.37
LVPW, diastole (mm)	1.79 ± 0.16	1.79 ± 0.16	1.81 ± 0.12	1.73 ± 0.09
LVPW, systole (mm)	2.76 ± 0.28	2.78 ± 0.20	2.84 ± 0.21	2.66 ± 0.15
Shortening fraction (%)	40 ± 3	35 ± 8	37 ± 2	44 ± 3
Ejection fraction (%)	67 ± 4	68 ± 5	67 ± 2	72 ± 3
Mitral flow				
Peak E (m/s)	1143 ± 178	979 ± 89	1127 ± 97	1080 ± 57
Peak A (m/s)	1040 ± 193	871 ± 53	855 ± 79	987 ± 71
E/A ratio	1.1 ± 0.1	1.1 ± 0.2	1.3 ± 0.1	1.1 ± 0.1
Pulmonary artery flow				
PAAT (m/s)	23.43 ± 1.64	13.95 ± 0.95*	22.14 ± 1.40	18.93 ± 1.80*^,†^
PAAT/Ejection time	0.32 ± 0.02	0.18 ± 0.02*	0.34 ± 0.02^†^	0.25 ± 0.03
Peak velocity (m/s)	1235 ± 71	978 ± 62*	1218 ± 54^†^	1111 ± 32
VTI (mm)	56 ± 2	36 ± 5*	53 ± 2^†^	46 ± 2

Rats consumed wine for 7 days before and 28 days after being injected with PAH-inducing MCT

Values are mean ± S.E.M

*C* Control, *MCT* Monocrotaline, *RARW* Reduced-alcohol red wine, *bpm* Heart rate, *IVS* inter-ventricular septum, *LVID* left ventricle internal diameter (mm) , *LVPW* left ventricle posterior wall, (mm), *Peak E* maximum velocity of mitral E wave (m/s), *Peak A* maximum velocity of mitral A wave (m/s), *PAAT* pulmonary artery acceleration time (m/s), *VTI* velocity time integral (mm)

*p < 0.05 (vs C); ^†^p < 0.05 (vs MCT); n ≥ 5 per group

#### Plasma lipid peroxidation markers (CD and TBARS) and antioxidant activities (SOD and catalase)

The concentration of CD increased in MCT vs controls (p < 0.01). Chronic and moderate treatment with RARW reversed the MCT-induced PAH -associated CD concentration (p < 0.01) as shown in Additional file 2: Figure S2. The concentration of TBARS increased in MCT vs controls (p < 0.01) and in MCT + RARW vs controls (p < 0.01). There was a tendency of reduction in the MCT + RARW group compared with the MCT groups but it was not significant. There were no significant changes in SOD and catalase activities after consumption of RARW, see Additional file 2: Figure S2.

#### Determination of cardiac fibrosis

No changes of cardiac fibrosis occurred following the injection of MCT (p > 0.05). Chronic and moderate treatment with RARW did not modify cardiac fibrosis in control or MCT treated rats (p > 0.05), as shown in Additional file 3: Figure S3.

### Discussion

In the present study, we used an established in vivo rat model of PAH to study the potential cardiovascular benefit of chronic and moderate consumption of RARW on cardiac dysfunction associated with PAH. Our data show, for the first time to our knowledge, that chronic and moderate consumption of RARW confers cardiovascular protection in an MCT-induced PAH model. Indeed, 28 days after MCT injection, PAH was characterised by an increase in RV hypertrophy and an alteration of the pulmonary artery flow. The daily treatment with moderate RARW in this PAH rat model reduced RV hypertrophy and improved pulmonary artery flow. Interestingly, this beneficial effect was associated with reduced lipid peroxidation.

Epidemiological studies consistently support the view that alcoholic beverages confer cardiovascular benefits in a J-shape curve fashion with maximal protection obtained for 1–2 glasses per day for women and 2–3 glasses per day for men [18]. There is no doubt that excessive consumption of alcohol produces detrimental effects on the cardiovascular system and other systems [19]. It is therefore critical to keep the alcohol content to a minimal intake so that cardiovascular benefit can be observed without any well-known side effects of the alcohol. To this end, we had previously established an animal rat model mimicking the human setting of chronic and moderate consumption of wine (equivalent to 1–2 glasses per day) to demonstrate that chronic and moderate consumption of RARW (6% alcohol by volume) could confer the same cardioprotective benefits against ischemia–reperfusion injuries as a regular wine (12% alcohol by volume) [4]. In the present study, we used the same protocol to demonstrate the cardiovascular benefit of RARW (thus limiting any potential side effects of the alcohol) in an in vivo animal model of pulmonary hypertension.

The potential benefit of chronic and moderate alcoholic beverage against pulmonary hypertension has been poorly studied in both clinical and experimental settings. Although a lower risk of chronic obstructive pulmonary disease is observed with moderate alcohol consumption in humans [6], no clear scientific evidence on the amount and duration of alcoholic beverage consumption is available [20]. Using both morphometric and echocardiographic measurements, our data provide solid evidence for a reduction of right ventricular hypertrophy associated with chronic and moderate consumption of RARW in a rat model of PAH. The use of echocardiography is a useful technique to measure pulmonary acceleration time as an alternative index to assess right ventricular systolic pressure in a non-invasive manner in PAH rats [21]. Currently, treatment in patients suffering from PAH has limited effect on mortality and the quality of life, does not cure the disease and only limits the progression of the disease [2]. Here, we show that chronic and moderate consumption of RARW could effectively reduce cardiovascular damage associated with PAH in a well-established preclinical model. Interestingly, epidemiological studies suggest that wine may confer superior health benefits compared to other alcoholic beverages, an effect that would be attributed, at least in part, to the various cardioprotective components that are present in the wine [5]. Unfortunately, very little experimental work has been performed in this field. Alcohol alone, given chronically at the concentration equivalent to 2–3 glasses of wine, does not confer cardioprotection against ischemia–reperfusion injury [4]. Other bioactive compounds such as resveratrol and melatonin, given chronically at the concentration found in wine, can protect against ischemia–reperfusion injury [22]. Grape juice, which contains similar phenolic compounds to wine, increases endothelial nitric oxide synthase activity, decreases right ventricular end diastolic pressure and lipid peroxidation in MCT-induced cor pulmonale [23]. These studies, combined with our present study, strongly support the knowledge that the alcohol may not be the main component in wine to account for the cardioprotective effect of chronic and moderate consumption of RARW. It would therefore be of interest to test whether moderate and chronic consumption of grape juice or red wine after complete removal of alcohol may still confer any cardiovascular benefit in a rat model of PAH.

The mechanisms responsible for the cardiac alterations in PAH still remain to be understood, although the presence of an oxidative stress may precipitate the harmful events leading to right ventricular cardiac hypertrophy (see review) [2]. Oxidative stress, a consequence of oxidants and antioxidants imbalance is associated with excess reactive species that may cause damage to biological systems, requiring repair and detoxification [24]. High consumption of antioxidants such as those found in red wine can decrease oxidative stress levels and contribute to health protection [25]. In the current study, moderate to chronic consumption of RARW significantly reduced CD similar to the findings already described in the literature with wine or dealcoholized wine [26, 27]. Significant reduction in oxidants after wine consumption suggests that moderate and chronic red wine consumption could protect against lipid peroxidation in the circulation [28, 29].

### Conclusion

In conclusion, chronic and moderate consumption of RARW decreased MCT-induced PAH, lessened RV hypertrophy and dysfunction, an effect which is associated with a reduction of lipid peroxidation. The results of this study suggest that chronic moderate consumption of RARW or its components may represent a promising new protective strategy to limit cardiovascular dysfunction in PAH.

## Limitations

Using MCT-induced PAH rats to study the effects of reduced-alcohol wine over a short period of time on potential cardioprotection, as well as on oxidative stress, is a relevant model. However, there are possible limitations of the study that need to be taken into consideration. The rats consumed RARW for 7 days prior to the MCT-injection. It is possible that this duration of treatment was insufficient to achieve maximal protection before the injection of MCT. Some studies have suggested that a cardioprotective effect of regular moderate consumption of alcohol might only be observed after 6 weeks of chronic drinking. To better mimic the natural conditions of wine drinking, the red wine could have been given at specific times during a 24-h period, by intragastric gavage. This would more closely mimic the human conditions where wine is consumed undiluted, often at specific times of the day, but would have increased the complexity of the study. Adding a control group of rats that consumed alcohol only might have provided information about the cardioprotective effect of the alcohol itself compared with the other components in wine. In our study, we used CD and TBARS assays to assess oxidative stress. These assays are still widely used due to their cost-effectiveness and ease of method. However, more accurate and sensitive (but often more costly and time-consuming) techniques to assess lipid peroxidation and oxidative stress are available.

## Supplementary Information

**Additional file 1: Figure S1.** Summarised experimental protocol of the study. Rats were divided into 4 groups: those who consumed water (Control), reduced-alcohol wine (RARW) and/or those who received an injection of PAH-inducing MCT. All rats consumed the respective beverages for 7 days before until 28 days after the MCT injection. MCT: Monocrotaline; RARW: Reduced-alcohol red wine; SOD: Superoxide dismutase; CD: Conjugated dienes; TBARS: Thiobarbituric acid reactive substances.

**Additional file 2: Figure S2.** The effect of chronic, moderate and reduced-alcohol red wine on oxidative stress parameters and antioxidants in controls and PAH rats. Rats were divided into 4 groups: those who consumed water (Control), reduced-alcohol wine (RARW) and/or those who received an injection of PAH-inducing MCT. All rats consumed the respective beverages for 7 days before until 28 days after the MCT injection. a Effect of moderate, chronic treatment of RARW on plasma conjugated dienes in MCT induced PAH. b Effect of moderate, chronic treatment of RARW on plasma TBARS in MCT induced PAH. c Effect of moderate, chronic treatment of RARW on plasma superoxide dismutase in MCT14induced PAH. d Effect of moderate, chronic treatment of RARW on plasma catalase in MCT induced PAH. C: Control; MCT: Monocrotaline; RARW: Reduced-alcohol red wine; CD: Conjugated dienes. Values are mean ± S.E.M. *p< 0.05 (vs C); †p<0.05 (vs MCT); n ≥ 5 per group.

**Additional file 3: Figure S3.** Determination of cardiac fibrosis in controls and PAH rats. Rats were divided into 4 groups: those who consumed water (Control), reduced-alcohol wine (RARW) and/or those who received an injection of PAH-inducing MCT. All rats consumed the respective beverages for 7 days before until 28 days after the MCT injection. a Effect of moderate, chronic treatment of RARW on cardiac fibrosis in MCT induced PAH. b Histology section of hearts (RV) stained with 0.1% Sirius red in picric acid to access cardiac fibrosis at 20X magnification. Data are from control rats and post MCT rats at day 28. C: Control; MCT: Monocrotaline; RARW: Reduced-alcohol red wine; RV: Right ventricle; LV: Left ventricle. Values are mean ± S.E.M. p>0.05; n ≥ 5 per group.

## Data Availability

The datasets used and/or analysed during the current study are available from the corresponding author on reasonable request. There are additional files. See the additional files.

## Abbreviations

PAH: Pulmonary arterial hypertension
RV: Right ventricle
RW: Red wine
RARW: Reduced-alcohol red wine
MCT: Monocrotaline
PAAT: Pulmonary artery acceleration time
VTI: Velocity–time integration
TAPSE: Tricuspid annular plane systolic excursion
CD: Conjugated dienes
TBARS: Thiobarbituric acid reactive substances
SOD: Superoxide dismutase
