# Supplementary figures and images for: Chronic and moderate consumption of reduced-alcohol wine confers cardiac benefits in a rat model of pulmonary arterial hypertension
Source: BMC Res Notes. 2021 Aug 23;14:324. doi: 10.1186/s13104-021-05738-x (PMC8381534; doi:10.1186/s13104-021-05738-x)

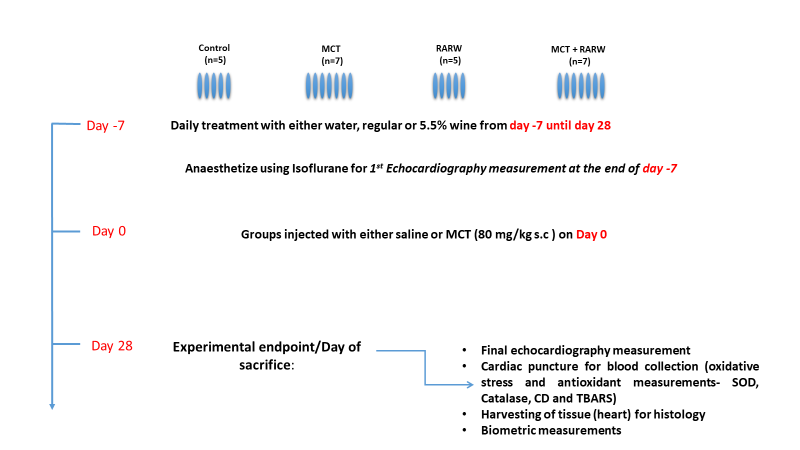

Supplement: Supplementary file 1 — Additional file 1: Figure S1. Summarised experimental protocol of the study. Rats were divided into 4 groups: those who consumed water (Control), reduced-alcohol wine (RARW) and/or those who received an injection of PAH-inducing MCT. All rats consumed the respective beverages for 7 days before until 28 days after the MCT injection. MCT: Monocrotaline; RARW: Reduced-alcohol red wine; SOD: Superoxide dismutase; CD: Conjugated dienes; TBARS: Thiobarbituric acid reactive substances. [file 13104_2021_5738_MOESM1_ESM.docx]
