# Supplementary material for: Chronic and moderate consumption of reduced-alcohol wine confers cardiac benefits in a rat model of pulmonary arterial hypertension
Source: BMC Res Notes. 2021 Aug 23;14:324. doi: 10.1186/s13104-021-05738-x (PMC8381534; doi:10.1186/s13104-021-05738-x)
Supplement: Supplementary file 2 [file 13104_2021_5738_MOESM2_ESM.docx]

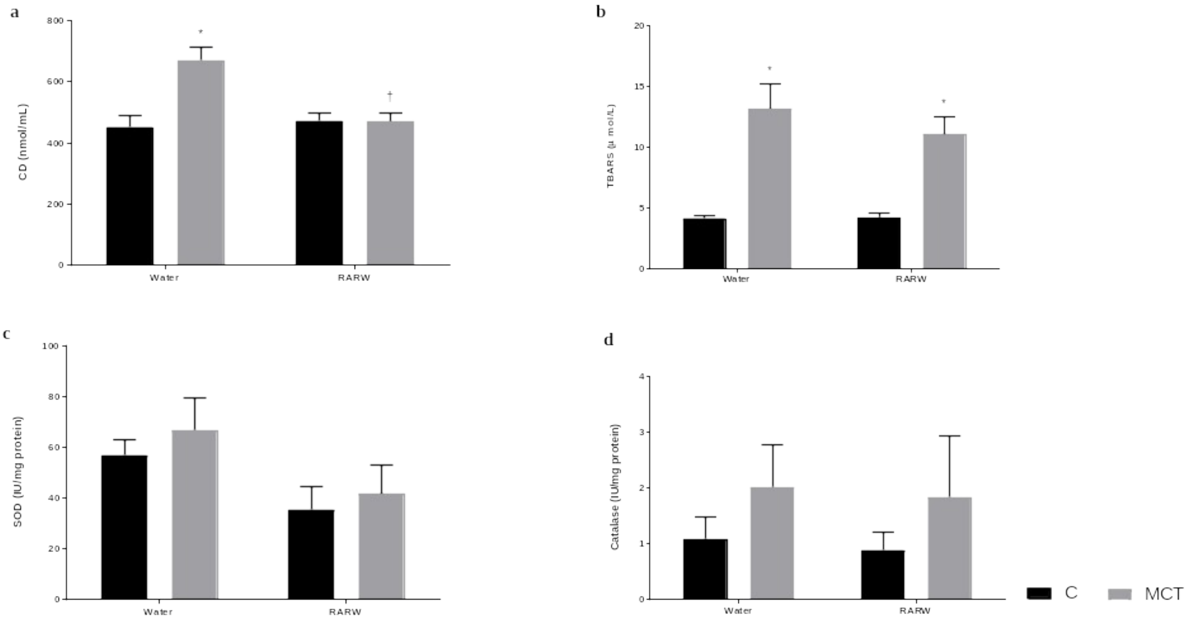
a
CD (nmol/mL)
0
200
400
600
800
*
†
Water
RARW
b
TBARS (μ mol/L)
0
5
10
15
20
*
*
Water
RARW
c
SOD (IU/mg protein)
0
20
40
60
80
100
Water
RARW
d
Catalase (IU/mg protein)
0
1
2
3
4
Water
RARW
C
MCT
