# Supplementary material for: Chronic and moderate consumption of reduced-alcohol wine confers cardiac benefits in a rat model of pulmonary arterial hypertension
Source: BMC Res Notes. 2021 Aug 23;14:324. doi: 10.1186/s13104-021-05738-x (PMC8381534; doi:10.1186/s13104-021-05738-x)
Supplement: Supplementary file 3 [file 13104_2021_5738_MOESM3_ESM.docx]

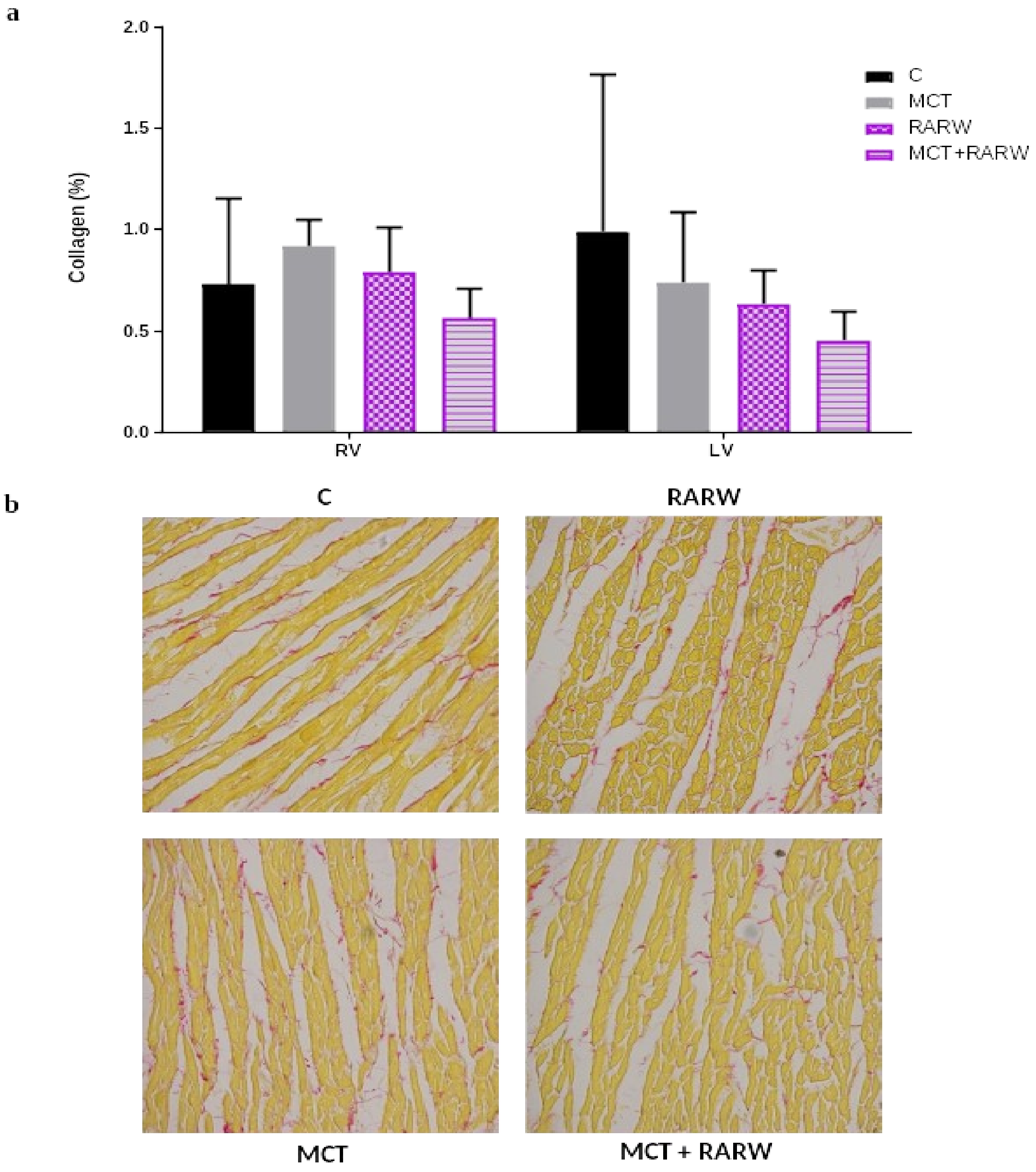
a
2.0
1.5
1.0
0.5
0.0
Collagen (%)
RV
LV
C
MCT
RARW
MCT+RARW
b
C
RARW
MCT
MCT + RARW
